# Pontomedullary junction as a reference for spinal cord cross-sectional area: validation across neck positions

**DOI:** 10.1038/s41598-023-40731-3

**Published:** 2023-08-19

**Authors:** Sandrine Bédard, Maxime Bouthillier, Julien Cohen-Adad

**Affiliations:** 1 NeuroPoly Lab, Institute of Biomedical Engineering, Polytechnique Montreal, Montreal, QC Canada; 2 Centre Hospitalier de l’Université de Montréal, University of Montreal, Montreal, QC Canada; 3 Functional Neuroimaging Unit, CRIUGM, University of Montreal, Montreal, QC Canada; 4 Mila - Quebec AI Institute, Montreal, QC Canada; 5 Centre de Recherche du CHU Sainte-Justine, Université de Montréal, Montréal, QC Canada

**Keywords:** Biomarkers, Neurology, Imaging techniques, Biomedical engineering, Spine structure

## Abstract

Spinal cord cross-sectional area (CSA) is an important MRI biomarker to assess spinal cord atrophy in various neurodegenerative and traumatic spinal cord diseases. However, the conventional method of computing CSA based on vertebral levels is inherently flawed, as the prediction of spinal levels from vertebral levels lacks reliability, leading to considerable variability in CSA measurements. Computing CSA from an intrinsic neuroanatomical reference, the pontomedullary junction (PMJ), has been proposed in previous work to overcome limitations associated with using a vertebral reference. However, the validation of this alternative approach, along with its variability across and within participants under variable neck extensions, remains unexplored. The goal of this study was to determine if the variability of CSA across neck flexions/extensions is reduced when using the PMJ, compared to vertebral levels. Ten participants underwent a 3T MRI T2w isotropic scan at 0.6 mm^3^ for 3 neck positions: extension, neutral and flexion. Spinal cord segmentation, vertebral labeling, PMJ labeling, and CSA were computed automatically while spinal segments were labeled manually. Mean coefficient of variation for CSA across neck positions was 3.99 ± 2.96% for the PMJ method vs. 4.02 ± 3.01% for manual spinal segment method vs. 4.46 ± 3.10% for the disc method. These differences were not statistically significant. The PMJ method was slightly more reliable than the disc-based method to compute CSA at specific spinal segments, although the difference was not statistically significant. This suggests that the PMJ can serve as a valuable alternative and reliable method for estimating CSA when a disc-based approach is challenging or not feasible, such as in cases involving fused discs in individuals with spinal cord injuries.

## Introduction

Spinal cord (SC) cross-sectional area (CSA) is a relevant biomarker to assess SC atrophy in diseases like multiple sclerosis (MS)^1,2^ and spinal cord injury^3–5^. Early neurodegeneration in MS requires high accuracy in detecting subtle atrophy for monitoring purposes^6^. Traditionally, CSA is measured at a specific rostro-caudal location based on vertebral levels to enable consistent comparisons through time in cross-sectional and longitudinal studies.

It is important to note that there is a distinction between vertebral levels and spinal levels. Inferring the neuroanatomic position of the spinal levels with the vertebrae can introduce inaccuracies, contributing to variability in CSA measurements^7^. Cadotte et al.^7^, showed that spinal segment positions differ across individuals and poorly overlap with vertebral bodies. A vertebral reference also does not account for variations in neck positioning within the MRI scanner. Spinal levels can be identified by segmenting the nerve rootlets, but it is a challenging task, and requires high resolution scans (about 0.6 mm^3^) and an expert rater. Having a more precise surrogate reference for spinal levels is important to link CSA measurements with the clinical outcome.

It is important to consider neck flexion and extension from an anatomical and biomechanical standpoint. The SC dura mater is a continuation of the cranial dura mater extending from the foramen magnum and the brainstem^8^. Head extension and flexion produce a change of direction of the nerve rootlets compared to a neutral posture where nerve rootlets run downwards. Neck flexion also elongates the SC, with its maximum between C2-T1 nerve rootlets^9^. Neck flexion and extension also produce movement of the spinal segments relative to vertebral segments: Bilston & Thibault^10^ showed during neck extension that C3 moves caudally of 5 mm vs. 2 mm at C5 compared to neutral positions.

A few have introduced surrogate neuroanatomic landmarks to overcome the limitation of the vertebral reference like the pontomedullary junction (PMJ)^7,11^ or the *conus medullaris*^12^.

We introduced in previous work an automatic pipeline to compute CSA at a distance from the PMJ in a cross-sectional study to reduce SC CSA inter-subject variability^13^. CSA averaged at the C2-C3 disc and CSA at 64 mm from the PMJ gave similar coefficients of variation (COV); no clear conclusion about the effect of the reference on CSA variability across individuals was found^13^. Further validation is necessary to evaluate the potential use of PMJ-based reference in the context of longitudinal studies.

The aim of this study is to investigate the relevance of the PMJ as an anatomical reference to mitigate intra-subject variability in CSA measurements in a longitudinal context. It involves (i) developing a pipeline to label the spinal segments using the nerve rootlets in T2-weighted (T2w) images, (ii) determining the variability of intervertebral discs and the PMJ across three neck flexion/extensions, and (iii) comparing CSA variability using the PMJ, traditional vertebral levels, and spinal segments as references.

## Material and methods

### Data acquisition

**Figure 1:**
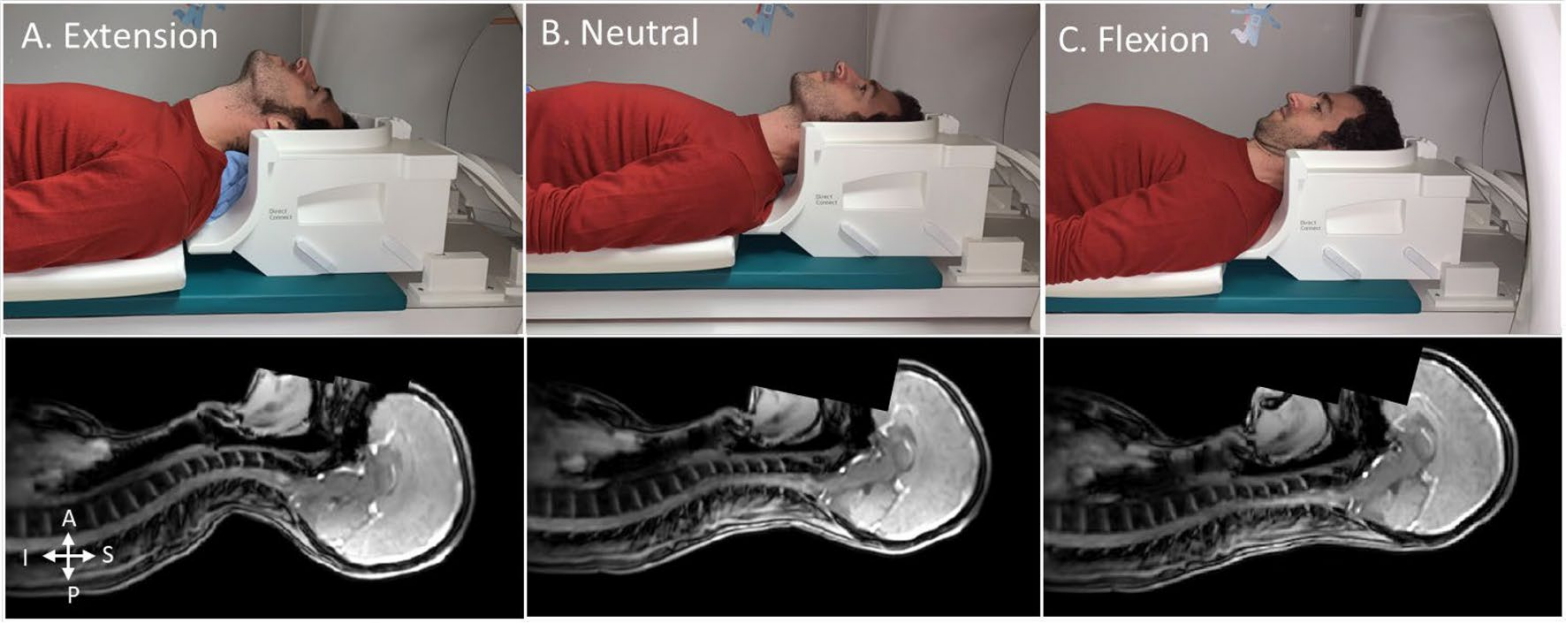
Example of the head positioning in the MRI’s head coil for neck extension (**A**), neutral (**B**) and flexion (**C**). The bottom row shows localizers. (One of the authors is in the scanner).

Ten healthy participants (Age: mean ± STD = 22.9 ± 1.2 years old; 3 females) were scanned using a Siemens 3T Prisma-fit with a 64 head-neck coil at three neck positions (extension, neutral, and flexion), as illustrated in Fig. 1. Informed consent was obtained from each participant and appropriate padding was used to maintain the head position and ensure comfort. The study was approved by the Comité d’éthique de la recherche du Regroupement Neuroimagerie Québec. All experiments were performed in accordance with the Declaration of Helsinki.

The field-of-view covered the top of the head down to at least the T1 vertebrae. A 3D sagittal T2w scan was acquired for each neck position with the following parameters: SPACE sequence, TR: 1.5 s, TE: 0.12 s, matrix: 72 × 384, in-plane resolution: 0.6 × 0.6 mm^2^, number of slices: 384, slice thickness: 0.6 mm, pixel bandwidth: 620 Hz/pixel).

### Data processing

The SC was automatically segmented using the Spinal Cord Toolbox (SCT) v5.4^14^ *sct_deepseg_sc*^15^ and the PMJ and vertebral levels were identified. The processing pipeline is available on GitHub (https://github.com/sct-pipeline/pmj-based-csa/releases/tag/r20230313) with its documentation (https://github.com/sct-pipeline/pmj-based-csa/tree/r20230313#readme).

#### Spinal segment labeling

**Figure 2:**
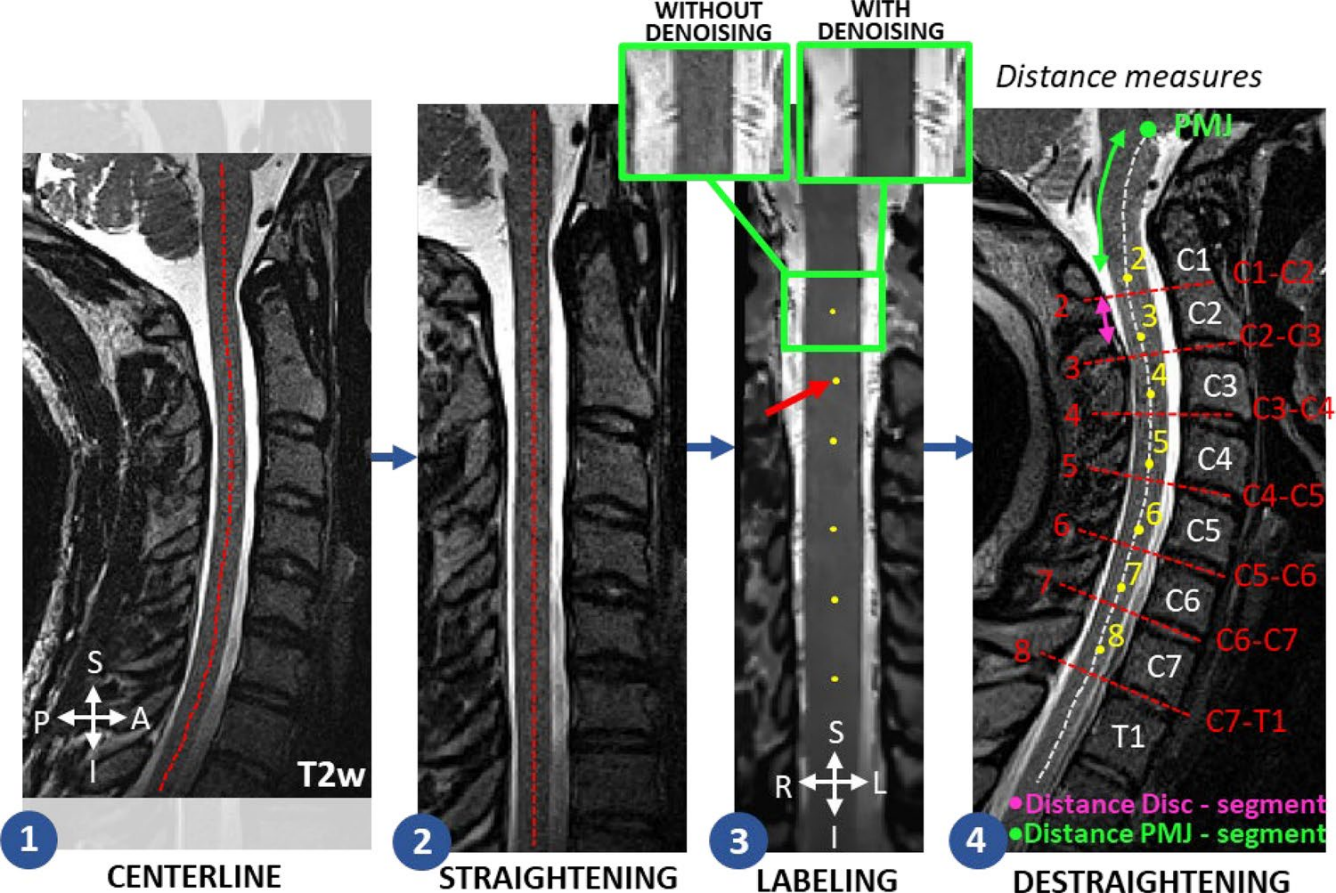
Spinal segment labeling pipeline. (1) SC centerline is detected, (2) SC is straightened, (3) denoised, and manually labeled in the coronal view. (4) The inverse transformation is applied on the labels. The distances between the PMJ and each spinal segment (green), and between each spinal segment and corresponding disc (pink) were computed.

Spinal segments were manually labeled on T2w images for each neck position by an expert rater (MB) using the spinal nerve rootlets to identify the mid spinal segment position (Fig. 2). The standardized procedure is available on GitHub (https://github.com/sct-pipeline/pmj-based-csa/tree/r20230313#manual-labeling-of-spinal-cord-rootlets-with-fsleyes).

Briefly, to label the spinal segments, we propose straightening the SC to enhance visualization of the spinal nerve rootlets, as the natural curvature of the SC can impede their clear identification in the coronal view. Non-local means adaptive denoising is applied to denoise the image^16^. In summary, the non-local mean filter leverages the similarity between distinct image patches to decrease noise while preserving details. Furthermore, it integrates the spatial and frequency information present in the image to adapt the denoising process. Spinal segment labels are then placed at the center (superior-inferior) of where the rootlet emerges from the SC on the corresponding axial slice in the center of the SC. The labeling convention is indicated in Fig. 2, and all spinal segments with visible nerve rootlets (except C1) are labeled for all neck positions and participants. The labels are finally brought back to the curved space with the inverse transform.

#### Distance with PMJ, spinal segment and discs

To estimate the position of the spinal segments using the PMJ and the discs as references, we computed two types of distances along the SC centerline for each participant and neck position: between the PMJ and each spinal segment (Fig. 2.4 green), and between each intervertebral disc and its corresponding spinal segment (Fig. 2.4 pink).

To assess the reliability of the spinal segment location estimation across the different neck positions, we computed the standard deviation (STD) across neck positions for each participant. The STD was computed for each method (PMJ, discs) and for each level (2 to 7). The STD was chosen to assess variability, as opposed to COV, because the absolute value of distances PMJ-spinal segment becomes larger for lower spinal segments, which would have influenced the COV when compared to disc-spinal segment distances. To assess if there was a significant difference between the STD of the estimated spinal segment positions measured with the PMJ and the discs, we conducted a paired two-way ANOVA test on the STD across neck positions (dependent variable) of the 10 participants. The independent variables were the methods (PMJ, discs) and levels (from 2 to 7). A significance level of *p-value* = 0.05 was set. We used the ANOVA test to detect a global effect of the independent variables rather than specific group differences. No post-hoc analysis was conducted.

#### CSA computation

**Figure 3:**
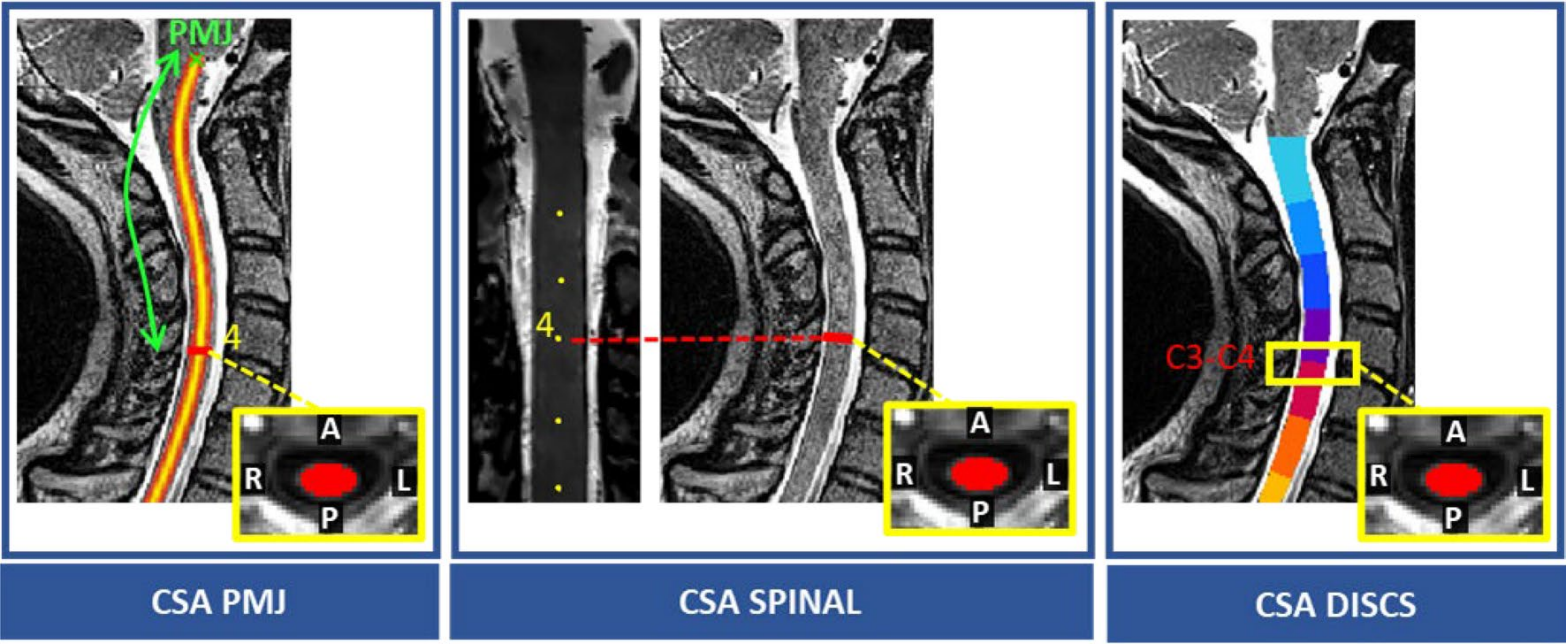
SC CSA computed with 3 references: (**A**) mean distance of each spinal segment from participant's PMJ, (**B**) each spinal segment, (**C**) each intervertebral disc.

SC CSA was computed using *sct_process_segmentation* and averaged across 3 slices using three references: CSA PMJ, CSA spinal, and CSA discs (Fig. 3). *CSA PMJ* was computed at the distance between the PMJ and each spinal segment, averaged across the three neck positions. That distance was subject-specific. The reason we chose to average the distance across neck positions is the following. The main idea of this paper is to validate a PMJ-based method^13^ as a reference for computing CSA instead of relying on intervertebral discs due to the known variability in the spatial correspondence between the spine and SC. As a reminder, the PMJ-based method relies on a set distance from the PMJ along the SC centerline. In the Bédard et al.^13^ paper, that distance was set to 64 mm, which corresponded to the distance to the C2-C3 disc, averaged across 804 adult participants. In the present study, instead of computing a distance between the PMJ and the C2-C3 disc, we estimate a distance between the PMJ and each spinal segment (manually identified with the nerve rootlets). That distance is calculated for each participant and for each neck position. Our hypothesis is that, for each participant, regardless of the neck extension, the PMJ-based CSA will give similar values (as opposed to the disc-based CSA, because the SC moves along the superior-inferior axis relative to the discs, depending on the neck position). To verify this hypothesis, we set a subject-specific distance between the PMJ and each spinal segment to be the average across the three neck positions. Then, we compute the CSA using that distance, and we evaluate the stability of that CSA measure. *CSA spinal* is computed at the spinal segment location for spinal levels C2 to C8, and *CSA discs* at the intervertebral discs C2-C3 to C7-T1.

To estimate the variability of CSA across neck positions, we computed the COV, instead of the STD in order to aggregate the results across participants and levels, since CSA varies across participants and across spinal levels^17^.

The COV across neck positions was computed for each participant, method and level as following:

$$COV[i]= STD(neck\, position\, n=3)/MEAN(n=3)$$

A paired 3-way ANOVA test was performed to evaluate if CSA computed using the 3 different references (PMJ, Spinal, and Disc) yielded equivalent measures per participant. The independent variables examined in our analysis were the methods (PMJ, Spinal, and Disc), levels, and neck positions. Note that the ANOVA test was specifically conducted on the CSA rather than on the COV, in order to be able to account for the neck position as an independent variable. Furthermore, due to the paired nature of the ANOVA test, COV was not relevant.

#### Neck angle

The angle of the SC was computed for each neck position in the right-left axis. The angle was computed between the C1-C2 and C4-C5 intervertebral discs (Fig. S1)^18,19^. The C4-C5 disc was chosen instead of the C6-C7 disc as done in the previous studies^18,19^ since one participant presented a smaller field-of-view in the superior inferior direction resulting in the absence of the C5-C6 and lower discs.

#### Quality control

After running analysis, the quality of all SC segmentations, vertebral labeling and PMJ labeling were visually assessed. Problematic segmentations (under-segmentation/leaking) and labels were identified in SCT's quality control report and manually corrected using ITK-SNAP. Disc labels were placed at the posterior tip of the disc for C1-C2 to C7-T1 and the PMJ was identified in the mid-sagittal plane. Corrections were added to the source dataset and the analysis was run again using the corrections instead of automatic labeling.

## Results

All data is accessible on OpenNeuro in the BIDS format^20^; manually corrected segmentations and PMJ, disc and spinal segment labels are also available: https://openneuro.org/datasets/ds004507/versions/1.0.1.

### Spinal segment labeling

**Figure 4:**
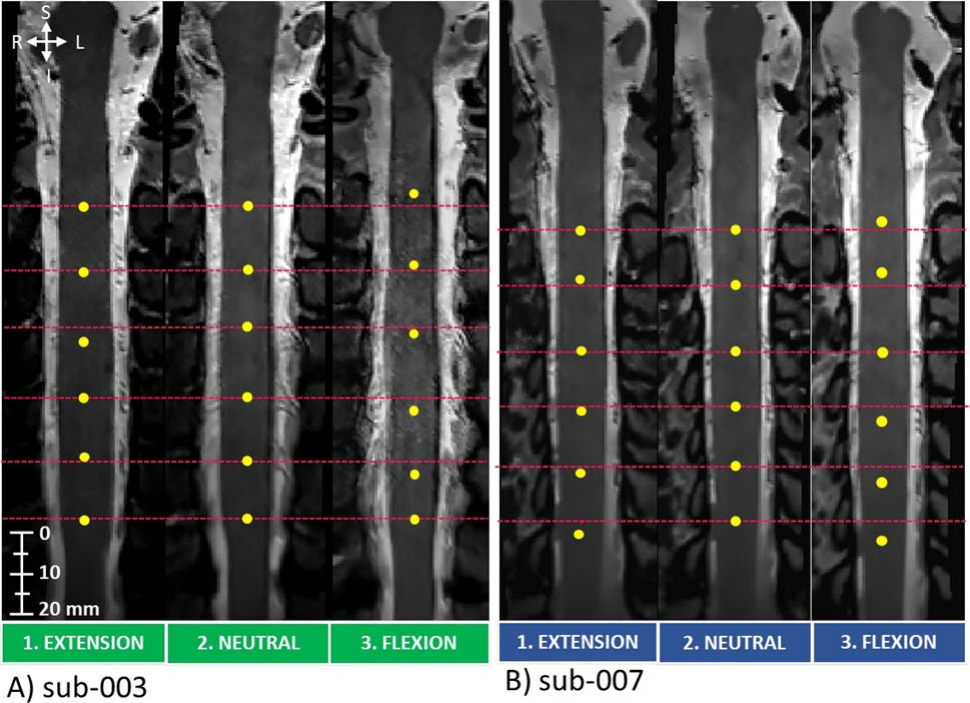
Example of challenging (**A**) and easy (**B**) spinal segment labeling in the straightened space in two representative participants. In the more challenging participants, motion and CSF flow likely contributed to the less apparent nerve rootlets.

Spinal nerve rootlets looked similar across neck positions in the straightened space per participant (Fig. 4). However, the center of spinal segments was more difficult to label in flexion due to motion artifacts, as seen in sub-003 (Fig. 4A.3) resulting in less defined nerve rootlets. In contrast, sub-007 (Fig. 4B) had good data quality with no motion artifacts and clear nerve rootlets.

### Distance with PMJ, spinal segment and discs

**Figure 5:**
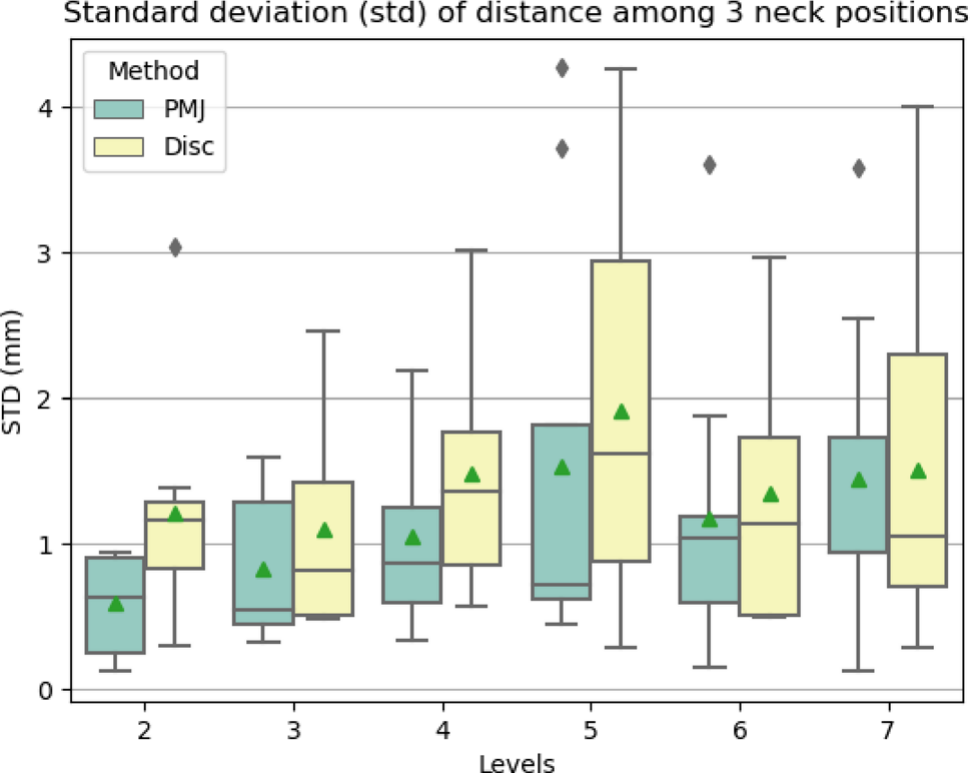
Boxplots (25–75 percentiles) of STD across neck positions per participant of the distance between the spinal segment and PMJ (green) and spinal segment and corresponding discs (yellow). Green triangles show the mean and gray diamonds outliers. No post-hoc analysis was performed beyond the ANOVA described in Table 2.

To estimate the position of the spinal segments using the PMJ and discs, we computed the distance PMJ-spinal segment and the distance disc-spinal segment. Next, we compared the reliability of estimating the spinal segments position across neck positions for each method (PMJ, discs). To assess this, we computed the STD of the distances across neck positions for both the PMJ and discs methods. Results are presented visually with a boxplot (Fig. 5), numerically (Table 1), and statistically (Table 2).

**Table 1:** STD of distance between spinal segments and PMJ or discs per level.

Levels	Distances STD (mm)	
	PMJ	DISC
	Mean ± std	Mean ± std
2	0.59 ± 0.31	1.19 ± 0.73
3	0.77 ± 0.51	1.14 ± 0.75
4	1.04 ± 0.63	1.37 ± 0.84
5	1.43 ± 1.42	1.85 ± 1.26
6	1.23 ± 1.00	1.35 ± 0.87
7	1.31 ± 1.10	1.52 ± 0.73
MEAN	1.06 ± 0.33	1.40 ± 0.26

**Table 2:** Paired Two-way ANOVA of STD of distance PMJ-spinal segment and disc-spinal segment across neck positions.

	F value	Num DF	Den DF	p-value
Method	0.795	1	8	0.399
Levels	2.618	5	40	0.0386*
Method x Levels	0.405	5	40	0.843

Significant values are in bold.

*p-value < 0.05. Method: PMJ or Disc. Levels: 2, 3, 4, 5, 6, 7. x: interaction. *F value*: F value of Fisher’s exact Test. *Num DF*: Degrees of freedom of numerator. *Den DF*: Degrees of freedom of denominator. *p-value*: p-value of Fisher Test.

Figure S2 shows scatterplots of the STD across neck positions of the distance PMJ-spinal segment (a) and the distance disc-spinal segment (b) for each participant.

Table 1 aggregates the STD across neck positions of the distance PMJ-spinal segment and disc-spinal segment between all participants and levels. The mean STD is lower when measuring from the PMJ than from the discs (1.06 ± 0.33 mm vs. 1.40 ± 0.26 mm).

To validate if there was a significant difference between the STDs across neck positions of the PMJ and discs method, we performed a paired two-way ANOVA test on the STD of distances across neck positions. Independent variables were methods (PMJ, Disc) and levels. Results are presented in Table 2. The analysis found no significant difference between the STD of PMJ and disc methods (*p-value* > 0.05), but a significant difference across levels (*p-value* = 0.0386). We did not conduct any further post-hoc analysis across levels. Our main focus was to investigate whether there were differences in the variability of the estimated spinal segment positions between the PMJ and discs-based estimation methods.

### CSA computation

**Figure 6:**
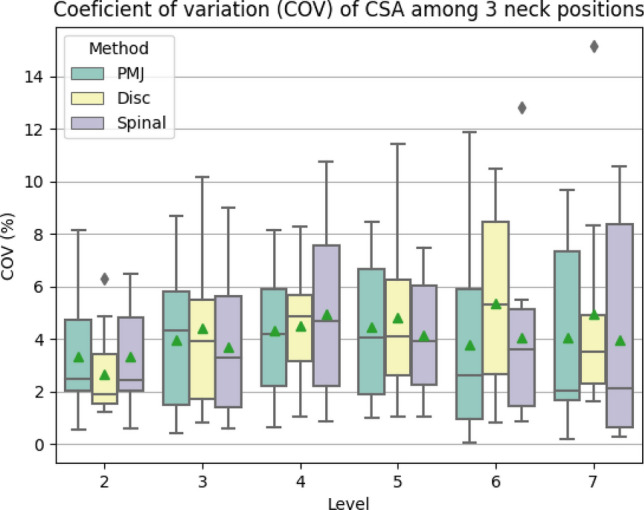
Boxplot of CSA COV per level based on discs C1-C2 to C7-T1, on spinal segments C2 to C8, and on the mean distance between the PMJ and each spinal segment. Green triangles represent the mean, and gray diamonds represent outliers.

CSA was computed from 3 anatomical references: PMJ, spinal segments, and discs. It was averaged on 3 slices per level and the COV across neck positions was computed for each participant, method, and level. Figure 6 shows boxplots of COV of CSA across neck positions for each method and level for a visual representation of the results. Mean CSA values across neck positions and participants for each method and level can be found in Table S1.

A scatterplot of COV for each participant and method is presented in Fig. S3. The mean CSA COV across participants, levels and methods is presented at Table 3.

**Table 3:** Mean COV of CSA per level across participants using PMJ, spinal segments and discs as a reference and averaged on 3 slices.

Levels	COV (%)		
	PMJ	Spinal	Discs
	Mean ± std	Mean ± std	Mean ± std
2	3.36 ± 2.44	3.32 ± 2.01	2.67 ± 1.74
3	3.97 ± 2.76	3.69 ± 2.76	4.43 ± 3.32
4	4.33 ± 2.53	4.97 ± 3.32	4.50 ± 2.39
5	4.45 ± 2.91	4.12 ± 2.26	4.82 ± 3.20
6	3.77 ± 3.64	4.04 ± 3.60	5.38 ± 3.62
7	4.07 ± 3.49	3.95 ± 4.10	4.95 ± 4.34
MEAN	3.99 ± 2.96	4.02 ± 3.01	4.46 ± 3.10

We performed a paired 3-way ANOVA test on SC CSA to assess statistical differences between methods (*PMJ*, *Spinal*, *Discs*), levels, and neck positions. Results are presented in Table 4. The ANOVA test results revealed no significant differences in CSA between methods or neck positions (*p-value* > 0.05). However, the test did indicate a significant difference in CSA across levels for all methods and neck positions (*p-value* = 0.000004). There was also a significant interaction between method, levels, and neck position on CSA (*p-value* = 0.0205), indicating that the relationship between methods and levels differed across neck positions.

**Table 4:** Paired Three-way ANOVA on SC CSA.

	F value	Num DF	Den DF	p-value
Method	2.413	2	18	0.118
Levels	26.269	3	27	0.000004*
Neck position	2.695	2	18	0.0947
Method x Levels	0.999	6	54	0.435
Method x Neck position	0.561	4	36	0.692
Levels x Neck position	0.823	6	54	0.557
Method x Levels x Neck position	2.131	12	108	0.0205*

Significant values are in bold.

*p-value < 0.05. Method: PMJ, Spinal, Discs. Levels: 2, 3, 4, 5. Neck position: Extension, Neutral, Flexion. x: interaction. *F value*: F value of Fisher’s exact Test. *Num DF*: Degrees of freedom of numerator. *Den DF*: Degrees of freedom of denominator. *p-value*: p-value of Fisher Test.

### Neck angle

We computed the neck angle between the C1-C2 and C4-C5 intervertebral discs (Fig. S1) to evaluate if the variation of neck angle had an impact on the observed variability between discs/PMJ and spinal segments. Table S2 displays participant neck angles for each neck position, with mean values of 18.45 ± 3.82° for extension, 11.14 ± 3.02° for neutral, and 4.79 ± 3.01° for flexion.

Table S3 presents the correlation between neck angle variation and spinal segment position variation (from PMJ or disc) between neck flexion and extension and Table S4 per level. Figure S4 presents a scatterplot of neck angle variation vs. variation of the distance spinal segment-PMJ and spinal segment-disc.

## Discussion

In this study, we evaluated the reliability of computing CSA using spinal segment positions, from intervertebral discs vs. from the PMJ in human participants across different neck positions (extension, neutral, flexion). The variability of the estimated spinal segments across neck positions using the PMJ vs. the discs did not differ significantly, although we noted a trend of lower variability (STD) for the PMJ method. Additionally, we compared SC CSA across 3 references (PMJ, intervertebral discs, and spinal segment) and we did not find any significant difference between CSA variability when using the PMJ as a reference, although the PMJ method yielded lower COV.

### Spinal segment manual labeling

Identifying nerve rootlets to label spinal segments is highly dependent on image quality, and even with high-resolution scans, it can be difficult, time-consuming and is not realistic for large scale studies. This highlights the need for a surrogate reference and further improvement of data acquisition quality. Flexion presented worse data quality of nerve rootlets due to increased artifacts caused by participants' movement (swallowing is difficult). The difficulty of accurately identifying nerve rootlets adds variability to distance and CSA measures. AI-based labeling methods could potentially increase the reliability of the labels in the future.

### Distance with PMJ, spinal segments and discs

The PMJ method was slightly more reliable than the disc-based method to estimate spinal segment position (STD PMJ: 1.06 mm vs. Disc 1.40 mm), although the difference was not statistically significant. However, it is important to consider the limitations of our study, such as the small number of participants and neck positions, as well as the variability in manual labeling (Fig. 5).

We observed higher disc vs. spinal segments discrepancies between neck positions than in a previous study^7^, possibly due to different acquisition parameters and the use of three positions in our study.

### CSA computation

The PMJ method has a 0.5% lower COV than the disc-based method on the CSA measures across neck positions, although that difference was not significant. A better COV helps reduce the minimum sample size required to detect atrophy in longitudinal studies, as demonstrated by Bautin et al.^21^. However, we still observed a 4% intra-subject variability between the neck positions, corresponding to a 3.08 mm^2^ difference at C5 (CSA of 77 mm^2^), corresponding to the cervical enlargement, and a 2.60 mm^2^ difference at C7 (CSA of 65 mm^2^).

The observed difference of 2.5–3 mm^2^, when compared to pathological cohorts, falls within the expected effect size for such cohorts. For example, in a systematic review, Casserly et al.^22^ reported an estimated atrophy rate of 1.78% per year in all MS patients, with a mean CSA at C2-C5 of 73.07 mm^2^, corresponding to a 1.3 mm^2^. It is important to consider that CSA measurements can be influenced by factors such as the segmentation method, acquisition parameters, scanner, and interindividual differences (sex, age)^13,21,23–25^.

This variability could be attributed to image quality, and over-segmentation of the SC due to residual segmentation errors (inclusion of spinal nerve rootlets). Depending on the neck position, the fanning in/out of the nerve rootlets at the proximity of the SC would be more or less tangential to the SC and could be more or less included in the automatic segmentation, resulting in variability of CSA across neck extensions. Some studies have also reported significantly smaller CSA and anterior–posterior diameter in neck flexion than neutral position with the elongation of the cord^26^, which could also contribute to the observed variability.

### Limitations & perspective

Limitations of this study include imprecise manual spinal segment labeling, which may explain why PMJ-based CSA had lower COV than when using spinal segments as a reference.

Measuring CSA based on the PMJ does not consider inter-subject SC length variability as discussed by Bédard et al.^13^. Hence, the PMJ-based approach, if not normalized, would be more suitable for longitudinal studies where participants would be their own controls. Metrics like cervical enlargement or total SC length could be suitable candidates for normalization, but further investigation is needed as no clear link of the spinal segments' location related to other anatomical references has yet emerged^27^.

Neck positioning can also vary in different clinical contexts^28^. For example, for SC compressions such as for patients with cervical myelopathy, studies have shown an increase in the severity of SC compression with the extension of the neck. It was found to be better correlated with clinical deficits than in a neutral position^18,29^. The variation of the spinal segments relative to the intervertebral discs is therefore important to consider when computing SC morphometrics such as CSA when varying the neck position. Furthermore, measuring at the precise location of the compression instead of averaging morphometrics across vertebral levels when using a distance from the PMJ could provide better monitoring of the compression and increase the sensitivity of myelopathy detection. Kinetic MRI is another example where the patient in a weight-bearing position can take various head flexion–extension positions; it can reveal abnormalities not shown by traditional supine or neutral MRI, mainly in SC injuries^30,31^.

## Conclusion

In this study, we investigated the use of the PMJ as an anatomical reference for computing SC CSA instead of relying on intervertebral discs. We compared the estimation of the spinal segments position and CSA measures using the PMJ vs. intervertebral discs as a reference. This comparison was performed across three neck positions using high isotropic resolution T2w scans. Our findings show that the PMJ method was slightly more reliable than the disc-based method to compute CSA at specific spinal segments, although the difference was not statistically significant. This suggests that the PMJ can serve as a valuable alternative and reliable method for estimating CSA when a disc-based approach is challenging or not feasible, such as in cases involving fused discs in individuals with SC injuries. Moreover, utilizing an anatomical reference based on the central nervous system could prove beneficial for studies investigating peripheral motor functions, dermatomes, and functional MRI studies.

### Supplementary Information

Supplementary Information.

## Data Availability

The datasets generated during and/or analyzed during the current study are available in the Spinal Cord Head Position MRI repository: https://openneuro.org/datasets/ds004507/versions/1.0.1.

## Abbreviations

COV: Coefficient of variation
CSA: Cross-sectional area
MS: Multiple sclerosis
PMJ: Pontomedullary junction
SC: Spinal cord
SCT: Spinal cord toolbox

## Code availability

The processing pipeline uses SCT v5.4 and is available on GitHub https://github.com/sct-pipeline/pmj-based-csa/releases/tag/r20230313 with its documentation: https://github.com/sct-pipeline/pmj-based-csa/tree/r20230313#readme.
