# Supplementary material for: Pontomedullary junction as a reference for spinal cord cross-sectional area: validation across neck positions
Source: Sci Rep. 2023 Aug 19;13:13527. doi: 10.1038/s41598-023-40731-3 (PMC10439961; doi:10.1038/s41598-023-40731-3)
Supplement: Supplementary file 1 — Supplementary Information. [file 41598_2023_40731_MOESM1_ESM.pdf]

# Supplementary material

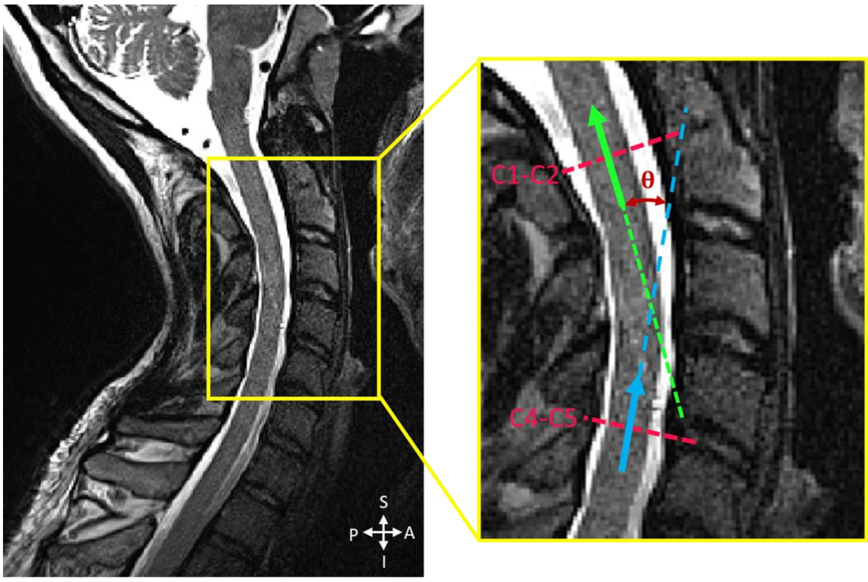


***Figure S1**. Neck angle measurement. The neck angle was computed in the right-left direction between the C1-C2 and C4-C5 intervertebral discs.*

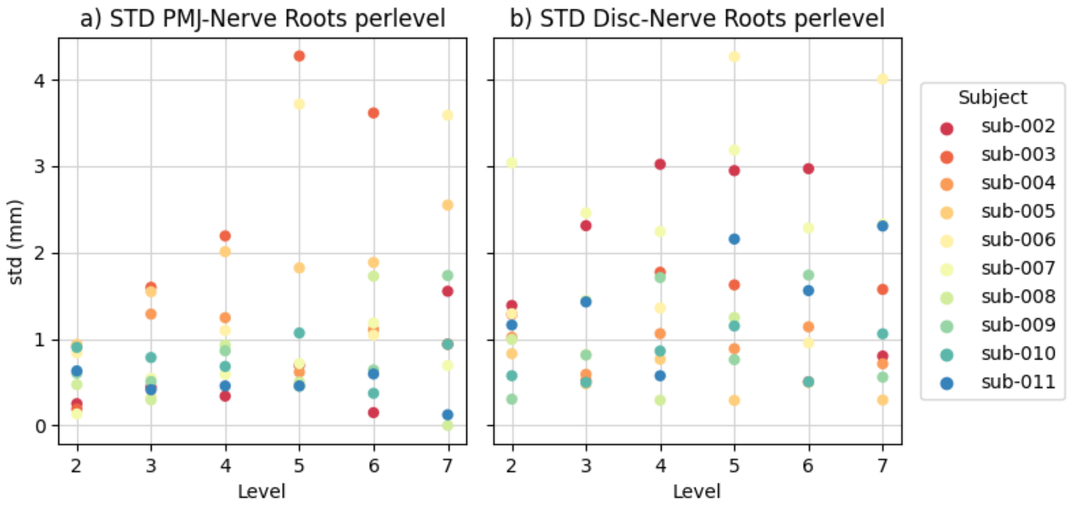


***Figure S2**. STD across neck positions of the distance between the PMJ and spinal segments **(a)** and between each disc and spinal segment **(b)** per participant.*

***Table S1.*** *SC CSA results across neck positions per method and level.*

| | ***CSA (mm²)*** | | |
|---|---|---|---|
| | ***PMJ*** | ***Spinal*** | ***Discs*** |
| ***Levels*** | ***mean ± std*** | ***mean ± std*** | ***mean ± std*** |
| 2 | 71.98 ± 4.58 | 72.31 ± 4.50 | 72.65 ± 4.70 |
| 3 | 71.66 ± 4.68 | 71.62 ± 4.74 | 70.75 ± 5.54 |
| 4 | 75.29 ± 5.62 | 74.71 ± 5.62 | 73.98 ± 5.09 |
| 5 | 77.17 ± 4.04 | 77.32 ± 4.03 | 77.00 ± 4.79 |
| 6 | 73.58 ± 5.17 | 73.81 ± 4.92 | 74.30 ± 4.55 |
| 7 | 65.04 ± 8.69 | 65.23 ± 8.38 | 64.50 ± 7.32 |
| **MEAN** | **72.45 ± 4.18** | **72.50 ± 4.09** | **72.20 ± 4.30** |

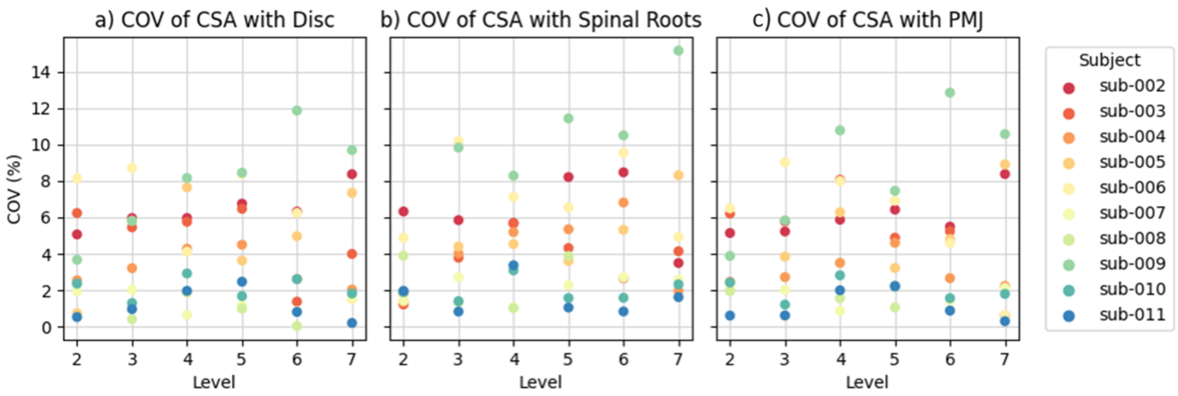


***Figure S3. a)*** *Scatterplot of COV of CSA per level based on intervertebral discs C1-C2 to C7-T1.* ***b)*** *Scatterplot of COV of CSA per level based on spinal segments C3 to T1.* ***c)*** *Scatterplot of COV of CSA per level based on the mean distance between the PMJ and each spinal segment. CSA is averaged on 3 slices.*

***Table S2.*** *Neck angle of the 3 neck positions per participant.*

| | ***Angles (˚)*** | | |
|---|---|---|---|
| ***Participants*** | ***Extension*** | ***Neutral*** | ***Flexion*** |
| sub-002 | 20.57 | 14.85 | 4.85 |
| sub-003 | 16.54 | 6.10 | -3.27 |
| sub-004 | 23.46 | 7.52 | 3.11 |
| sub-005 | 28.84 | 14.46 | 4.01 |
| sub-006 | 20.02 | 14.18 | 8.99 |
| sub-007 | 11.90 | 9.34 | 2.97 |
| sub-008 | 17.63 | 14.46 | 8.34 |
| sub-009 | 16.46 | 12.53 | 9.02 |
| sub-010 | 13.42 | 6.54 | 7.82 |
| sub-011 | 15.68 | 11.46 | 2.11 |
| **MEAN** | **18.45** | **11.14** | **4.79** |
| **STD** | **3.82** | **3.02** | **3.01** |

***Table S3.*** *Correlation between neck angle variation and variation of distance of the spinal segment and PMJ/discs between neck flexion and extension.*

| | ***Δ angles*** | ***level*** | ***Δ distance_pmj*** | ***Δ distance_disc*** |
|---|---|---|---|---|
| ***Δ angles*** | 1 | 0.024 | -0.503 | 0.164 |
| ***level*** | - | 1 | -0.419 | -0.096 |
| ***Δ distance_pmj*** | - | - | 1 | - |
| ***Δ distance_disc*** | - | - | - | 1 |

*Δ: variation between neck flexion and extension position*

***Table S4.*** *Correlation between neck angle variation and distance of the spinal segment variation between neck flexion and extension for PMJ and discs per level.*

| | ***Correlation with Δ angle*** | |
|---|---|---|
| ***Level*** | ***Δ distance_pmj*** | ***Δ distance_disc*** |
| 2 | **0.084** | -0.709 |
| 3 | -0.897 | **0.796** |
| 4 | **-0.426** | -0.454 |
| 5 | -0.657 | **0.584** |
| 6 | **-0.069** | -0.594 |
| 7 | -0.518 | 0.321 |

***BOLD****: minimum absolute correlation.*

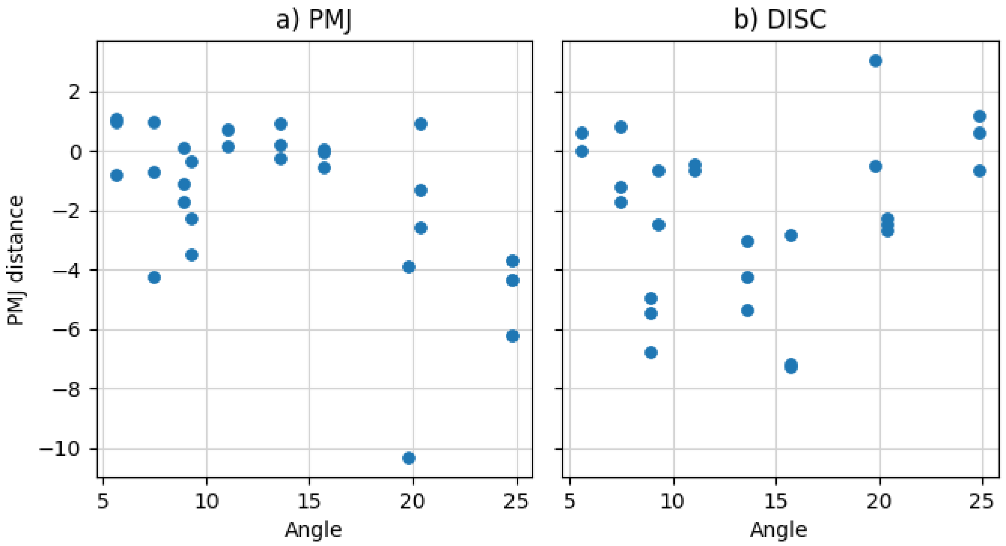


***Figure S4***. *Each dot corresponds to a participant position and angle. For each participant, we calculated the angle between the spinal cord centerline at C2-C3 and C4-C5 levels, and we took the difference of that angle between the neck extension and flexion: this is what is represented in the abscissa. The ordinate represents the difference of distance, between extension and flexion, between the PMJ and the corresponding spinal segment (PMJ-segment C2 ; PMJ-segment C3, etc.)* ***(a)*** *and the difference of distance between the corresponding disc and the corresponding spinal segment (disc C2 - segment C2; disc C3 - segment C3, etc.)* ***(b)****.*
